# 111 oriented gold nanoplatelets on multilayer graphene as visible light photocatalyst for overall water splitting

**DOI:** 10.1038/ncomms11819

**Published:** 2016-06-06

**Authors:** Diego Mateo, Iván Esteve-Adell, Josep Albero, Juan F. Sánchez Royo, Ana Primo, Hermenegildo Garcia

**Affiliations:** 1 Instituto de Tecnología Química, Universitat Politècnica de València-Consejo Superior de Investigaciones Científicas, Avenida de los Naranjos s/n, 46022 Valencia, Spain; 2 ICMUV, Instituto de Ciencia de Materiales, Universidad de Valencia, PO Box 22085, 46071 Valencia, Spain

## Abstract

Development of renewable fuels from solar light appears as one of the main current challenges in energy science. A plethora of photocatalysts have been investigated to obtain hydrogen and oxygen from water and solar light in the last decades. However, the photon-to-hydrogen molecule conversion is still far from allowing real implementation of solar fuels. Here we show that 111 facet-oriented gold nanoplatelets on multilayer graphene films deposited on quartz is a highly active photocatalyst for simulated sunlight overall water splitting into hydrogen and oxygen in the absence of sacrificial electron donors, achieving hydrogen production rate of 1.2 mol_H2_ per g_composite_ per h. This photocatalytic activity arises from the gold preferential orientation and the strong gold–graphene interaction occurring in the composite system.

## Abstract (web summary)

A plethora of photocatalysts have been investigated in order to obtain solar fuels but the photon-to-hydrogen molecule conversion is generally remains low. Here the authors show that 111 facet oriented gold nanoplatelets on multilayer graphene films is an active photocatalyst for overall water splitting.

## Graphical abstract

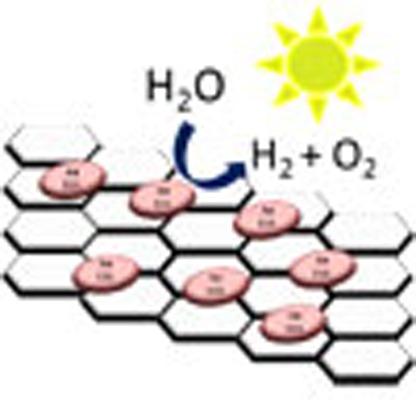

In the context of converting solar light into fuels there is a considerable interest in developing efficient photocatalysts for overall water splitting that could generate hydrogen in the absence of sacrificial electron donors under visible light (*λ*>400 nm) irradiation^1^,^2^,^3^,^4^,^5^. Besides TiO_2_ and other inorganic semiconductors^6^, the use of graphenes (G) as additive or as photoactive component for the photocatalytic production of solar fuels is an active area of research^7^,^8^,^9^,^10^,^11^,^12^. G as photocatalyst offers the advantages of sustainability, when obtained from biomass^13^,^14^,^15^,^16^,^17^, and the possibility of certain bandgap control either by doping or postsynthetic modification^18^,^19^. Graphitic carbon nitride (g-C_3_N_4_) is one of the most active photocatalysts based on a non-metallic semiconductor^20^,^21^. It has been reported that g-C_3_N_4_ containing a Pt as co-catalyst can generate hydrogen on solar light irradiation^22^. However, reports of hydrogen generation by Pt/G-C_3_N_4_ as photocatalyst use tertiary amines as sacrificial electron donors and, as far as we are aware, no overall water splitting with visible or solar light has been reported so far either for G or for G-C_3_N_4_ in the absence of sacrificial electron donors.

In the present manuscript we describe that 111 facet-oriented Au nanoplatelets supported on multilayer G (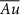/*ml*-G; 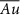 meaning 111 facet-oriented Au nanoplatelets and *ml*-G meaning multilayer graphene) is an efficient photocatalyst for the overall water splitting in the absence of any additive reaching on simulated sunlight H_2_ production rates about 0.9 mol_H2_ per g_composite_ per h and apparent quantum yields of 0.08%. In addition, 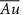/*ml*-G exhibits among the highest photocatalytic activity reported under visible illumination due to 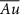 plasmon band excitation with a H_2_ production rate above 100 mmol_H2_ per g_composite_ per h on ultraviolet-filtered simulated sunlight. In the case of TiO_2_, it has been found that the lack of visible light photoresponse can be overcome by using Au nanoparticles (NPs) as light harvester and co-catalyst^23^,^24^. Strong evidence supporting that irradiation at the Au NP surface plasmon band introduces photocatalytic activity in TiO_2_ was obtained from the coincidence of the absorption spectrum of Au/TiO_2_ and photoresponse in the visible light^25^. Similarly, in the present case, a plasmon band photosensitization is observed for oriented 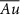/*ml*-G. Evidence is presented supporting that the unprecedented photocatalytic activity of 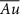/*ml*-G arises from the occurrence of a strong Au-G interaction derived from the unique features of the preparation procedure.

## Results

### Material preparation


**Figure 1:**
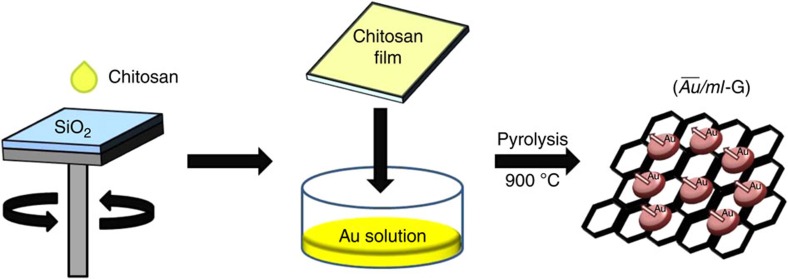
Preparation of 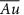/*ml*-G films. The films were prepared by spin coating on clean substrate a chitosan solution that is subsequently immersed in HAuCl_4_ solution and pyrolysis at 900 °C under inert atmosphere.

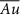
/*ml*-G films were prepared in a single step by pyrolysis at 900 °C under inert atmosphere of chitosan films of nanometric thickness containing HAuCl_4_. Chitosan is obtained by deacetylation of natural chitin that is the main compound of insect and crustacean skins^26^. It has been previously found that chitosan forms uniform defect- and crack-free films of subnanometric rugosity on arbitrary substrates and that pyrolysis of these films result in the formation of single, few or multilayer G (ref. 27). Of relevance in the present case is also the known ability of chitosan and other biopolymers to adsorb metals in aqueous phase by complexation of the metal ions by the polysaccharide fibrils^28^. In the present case, HAuCl_4_ was adsorbed on chitosan films on quartz before proceeding to the formation of G. Figure 1 summarizes the procedure followed for preparation of 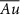/*ml*-G.

The Au content on *ml*-G film can be controlled in the range from ng to μg per cm^2^ by varying the concentration of HAuCl_4_ in the aqueous solution used for chitosan film impregnation from 10 to 1,000 μM. Methods section contains the exact amounts used in the preparation of the samples under study. Recently, the present procedure for the preparation of 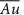/G has been reported by us^29^. Atomic force microscopy (AFM) measurements show that the thickness of the *ml*-G film of the samples under study is about 20 nm. The use of *ml*-G films is convenient to increase light absorption by G. It is known that single-layer G films have almost complete transparency (>99% transmittance), while the transparency decreases significantly for two layers (transmittance about 95%), four layers (transmittance about 85%) and multilayer (lesser transmittance). The present samples of 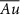/*ml*-G are visually black films with metallic reflection.

For the sake of comparison besides 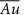/*ml*-G at three Au contents, a sample of unoriented *Au*/*ml*-G was also prepared by polyol reduction method of Au(III) and subsequent adsorption of the resulting unoriented Au NPs on ml-G (*Au*/*ml*-G) obtained by exfoliation of carbon residue of chitosan powder after pyrolysis. Other sample for control purposes consisted in *ml*-G films on quartz prepared by 900 °C pyrolysis of chitosan films in the absence of HAuCl_4_.

The Au content after pyrolysis of 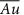/*ml*-G was determined by Au dissolution using aqua regia and subsequent quantitative inductively coupled plasma optical emission spectroscopy (ICP-OES) analysis of the resulting liquor, being the analytical data in good agreement with the values considering the complete adsorption by the chitosan film of the HAuCl_4_ present in the impregnation step. As previously commented, the use of chitosan in water purification is based on its ability to adsorb metals from water^30^,^31^. For the present study, three 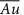/*ml*-G films with Au content from 0.2 to 13.5 μg_Au_ cm^−2^ were prepared.

**Figure 2:**
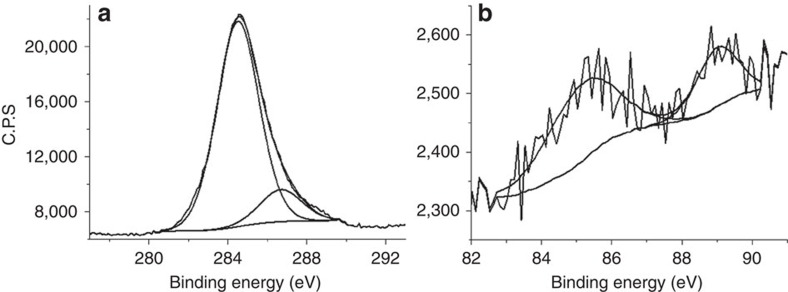
XPS measurements. (**a**) C 1s peak showing the deconvolution into two types of C, graphenic and C bonded to oxygen, and (**b**) Au 4f_7/2_ and 4f_5/2_ peaks showing the presence of this element in the 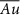/*ml*-G surface.

Formation of *ml*-G in the pyrolysis process was confirmed by Raman and X-ray photoelectron (XP) spectroscopies, whose spectra coincide with earlier precedents^32^. Specifically, in Raman spectroscopy the graphitic (G) and defect (D) bands at about 1,600 and 1,350 cm^−1^ were also recorded for 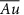/*ml*-G with *I*_G_/*I*_D_ ratio of 1.13 (Supplementary Fig. 1). The XPS peaks of C 1s and Au 4f and their corresponding deconvolution showing the contribution of the individual components are presented in Fig. 2. The C1 s peak has a major component at 284.5 eV (86%) corresponding to graphenic carbons accompanied by minor contributions at 286.7 eV (13%) due to carbons containing oxygen atoms in their coordination. The presence of Au was also detected by XPS that shows the 4f peaks corresponding to Au(0) at binding energies of 85.5 and 89.09 eV (0.019 atomic %), appearing about 1.4 eV shifted to higher values with respect to the binding energy of bulk Au appearing at 84.1 and 87.7 eV confirming the strong interaction of the nanoparticles with the graphene support^33^. It is worth noting that despite the fact chitosan has been reported to render N-doped G after pyrolysis, no peak corresponding to residual N is observed in the XPS. This behaviour has been described before and has been attributed to the influence of the metal NPs-G interaction, which heals defects in G and, particularly, removes completely N atoms leaving a minor proportion of oxygenated functional groups^34^.

**Figure 3:**
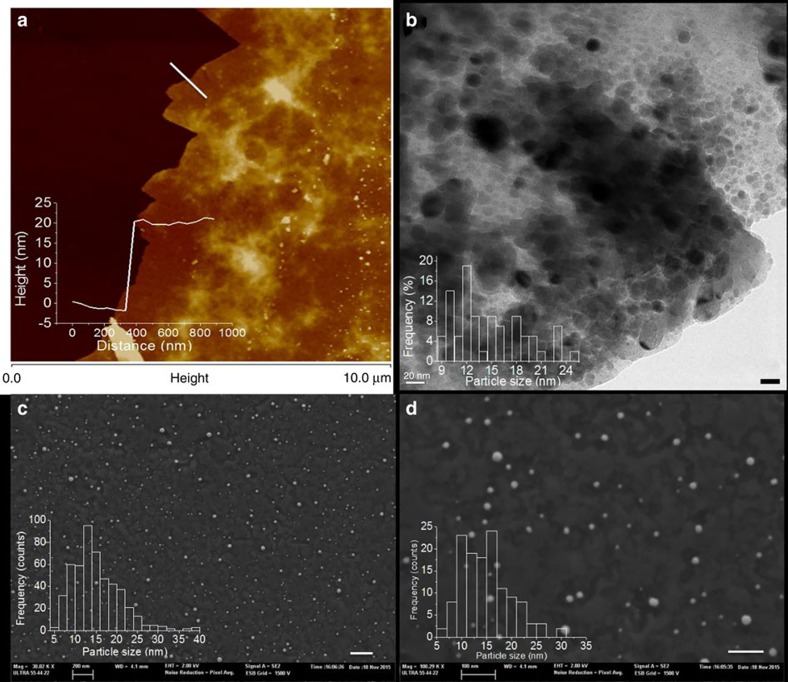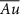/*ml*-G images. AFM frontal view (**a**), TEM micrograph (scale bar, 20 nm) (**b**) and FESEM images (scale bar 20 nm) at two different magnifications (**c**,**d**) of a 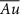/*ml*-G film (1 μg Au per cm^2^) (scale bars of 200 and 100 nm for **c** and **d**, respectively). Inset in **a** shows the thickness of the *ml-G* film across the white line, while insets in **b**–**d** are the histograms showing Au nanoplatelet lateral size distribution.

The morphology and size of Au NPs was determined by field emission scanning electron microscopy (FESEM) and atomic force microscopy (AFM) images. The morphology of Au as nanoplatelets and their lateral dimensions ranging from 5 to 25 nm was established by counting a statistically relevant number of particles (Fig. 3). AFM allows determining with accuracy the height of the nanoplatelets between 2 and 4 nm (Supplementary Fig. 2).

**Figure 4:**
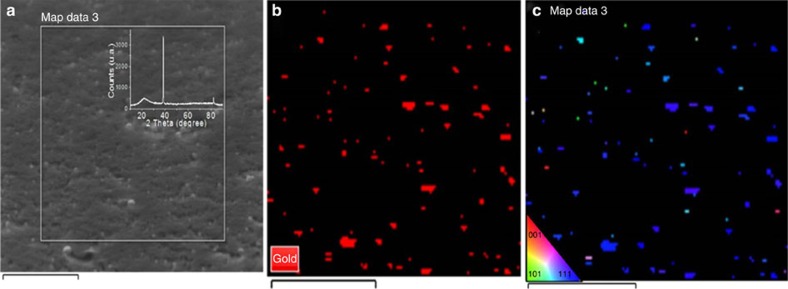
Electron microscopy images of 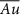/*ml*-G. (**a**) FESEM image of 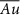/*ml*-G. (**b**) EDX image of the square indicated in the left FESEM image mapping the presence of Au. (**c**) Image of Au nanoplatelets showing 111 facet orientation in blue (scales bar, 500 nm in all cases). The inset shows the colour codes corresponding to other facet orientations. Scale bars, 500 nm.

The preferential 111 facet orientation of 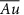/*ml*-G was confirmed by X-ray diffraction of the film (inset Fig. 4a). While Au NPs show five major diffraction peaks in X-ray diffraction (Supplementary Fig. 3), in the case of 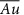/*ml*-G only the peaks corresponding to 111 and 222 diffractions are observed, indicating that these nanoplatelets have a preferential 111 facet orientation. The broad diffraction peak at 2Θ about 24° corresponds to the diffraction of *ml*-G. Further experimental evidence supporting the claim of 111 facet orientation was obtained by an electron backscattering diffraction (EBSD) technique in scanning electron microscopy (Fig. 4)^35^,^36^. In this case, orientation maps were collected by stepping the electron beam across the surface of the sample and indexing the resulting diffraction patterns. energy dispersive X-ray spectroscopy (EDX) image of 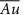/*ml*-G films shows the location of Au nanoplatelets and comparison of this image with that obtained by electron diffraction corresponding to the 111 diffraction shows a remarkable coincidence. Quantitative comparison of the EDX and 111 diffraction images using the ImageJ programme shows that about 90% of the particles present in the EDX image have 111 facet orientation.

Additional images supporting 111 facet orientation of Au nanoplatelets were obtained by transmission electron microscopy (TEM) after detaching the 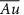/*ml*-G films from the quartz substrates by mechanical polishing, dimpling grinding and final Ar ion bombardment (Supplementary Fig. 4). The images show the presence of Au as thin nanoplatelets. These nanoplatelets present preferential 111 facet orientation as shown by the electron diffraction pattern with the expected interplanar distance of 0.23 nm (ref. 37). High-resolution-TEM (HR-TEM) images (Supplementary Fig. 4b) show the presence of crystalline graphene.

### Photocatalytic tests

**Figure 5:**
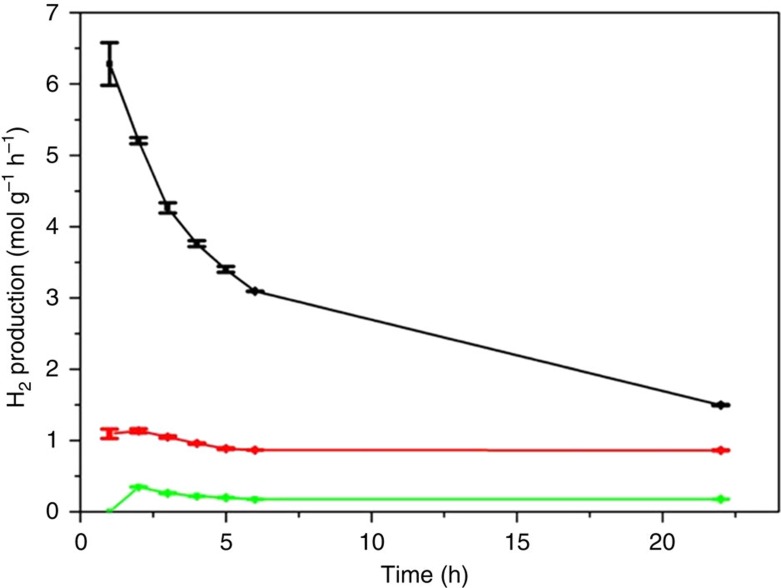
Temporal evolution of hydrogen production rate. Experiments were carried out under ultraviolet–visible irradiation in the presence (black line) and in the absence (green and red lines) of TEOA as sacrificial agent using a 2 × 2-cm^2^ 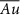
*/ml*-G film (Au content 1 μg cm^−2^, total photocatalyst mass including *ml*-G 4.25 μg cm^−2^). The red line corresponds to the experiment in the absence of TEOA without any filter, while the green line was obtained irradiating through an ultraviolet cutoff filter. The plots show the H_2_ production with the estimated errors (calculated as the square root of the sum of (a-ā)^2^, being a the value of the data set and ā the mean of the data set, divide by the number of data points) referred to the total Au plus *ml*-G photocatalyst amount (see Methods section for the estimation of *ml*-G weight).

Initial studies on the photocatalytic activity for H_2_ generation of 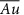/*ml*-G were carried out by irradiating with a 300-W Xe lamp having quasi constant irradiance at all wavelengths in the ultraviolet and visible region (see Supplementary Fig. 5 for the lamp emission spectrum). 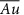/*ml*-G films coating a quartz square were placed on a sealed photoreactor (see also Supplementary Fig. 5 for a photoreactor photograph) and covered with an aqueous solution containing triethanolamine (TEOA) as sacrificial electron donor, observing the evolution of H_2_ gas determined by micro-GC (see Methods section for experimental details). Figure 5 shows the temporal evolution of H_2_ production rate in the presence of TEOA.

As it can be seen in Fig. 5, using TEOA as sacrificial electron donor agent the H_2_ production rate decreases along the irradiation time, a fact that can be due to the decrease of TEOA concentration and by the accumulation of by-products derived from this tertiary amine that can act as light filters and/or photocatalyst poison. In the rest of the study, no sacrificial electron donor was used and all the experiments were performed using MilliQ water (pH 6).

Importantly, H_2_ evolution was also observed in the absence of TEOA (see red line, Fig. 5), although the production rate was decreased by a factor of 5.5 times indicating that in the absence of TEOA the consumption rate of positive h^+^ holes is the limiting kinetic step. In accordance with the absence of the sacrificial electron donor, besides H_2_, evolution of O_2_ was also observed in the absence of TEOA at a rate that agrees with the expected stoichiometry for overall water splitting, with some differences at short irradiation times. This deviation from the 2:1 stoichiometry has been previously observed in the literature and attributed to changes in the oxygen content of the photocatalyst or to oxygen adsorption on the photocatalyst^38^. It could also be that the presence of some minor impurities acting at initial irradiation times as sacrificial electron donors could be at the origin of this misbalance that is not observed as the reaction progresses. In the absence of sacrificial electron donor, H_2_ production rate along the irradiation time was varying from 1.2 to 1 mol_H2_ per g_composite_ per h in 24 h. The main decrease in H_2_ production rate took place during the first 5 h of irradiation; afterwards, the production rate remained practically constant within 24 h. Similar behaviour can be observed for the ultraviolet-filtered measurement (Fig. 5, green line). This relatively minor decrease in the photocatalytic activity in 24 h could be due to deactivation by the presence in the photoreaction of some O_2_. Since the overall water splitting produces simultaneously H_2_ and O_2_, the quenching due to the residual presence of O_2_ at initial irradiation times is low and does not interfere in the reaction. However, at longer times, increasing amounts of residual O_2_ remaining in the photoreactor could inhibit partially the H^+^ reduction until a stationary production is reached.

**Figure 6:**
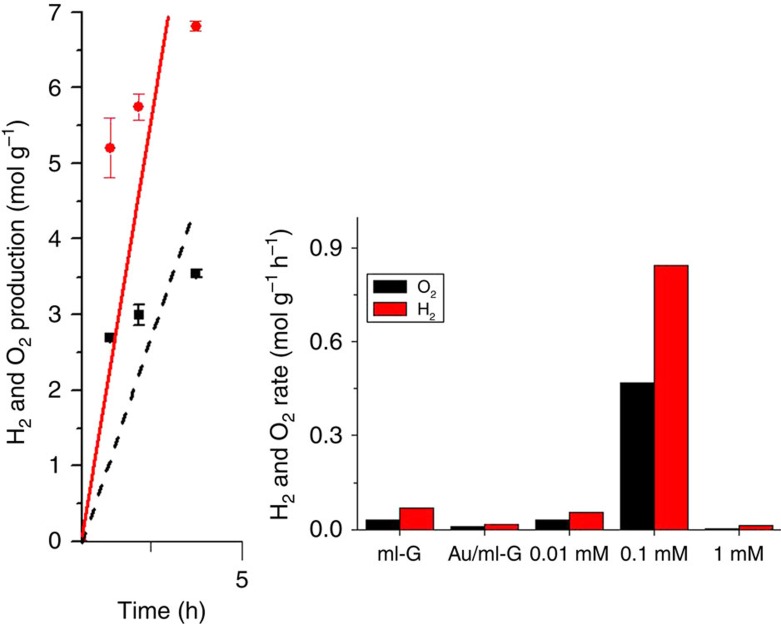
Photocatalytic H_2_ and O_2_ production. Temporal evolution of H_2_ and O_2_ evolution in mols per total mass of photocatalyst on irradiation of Ar-purged MilliQ water in contact with 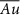
*/ml*-G (Au content 1 μg × cm^−2^, total photocatalyst content 4.25 μg cm^−2^). The plot shows the H_2_ and O_2_ production with the estimated s.d. (calculated as the square root of the sum of (a-ā)^2^, being a the value of the data set and ā the mean of the data set, divide by the number of data points). Photocatalytic H_2_ and O_2_ production rate of various materials for overall water splitting under the same conditions: *ml*-G (G content 3.25 μg cm^−2^), *Au/ml*-G (unoriented, Au content 30.6 μg, total photocatalyst content 30.6 mg) and 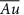
*/ml*-G (Au content 1 μg × cm^−2^, total photocatalyst content 4.25 μg cm^−2^).

The influence of Au loading on the photocatalytic activity of 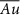/*ml*-G was determined by comparing H_2_ production rate of three samples prepared under the same conditions, but differing in about one order of magnitude the Au content (0.2, 1.0 and 13.5 μg cm^−2^, while the G content was constant at 3.25 μg cm^−2^). The results are also presented in Fig. 6, where the temporal profile of H_2_ and O_2_ evolution for the most efficient photocatalyst is also presented. Supplementary Fig. 6 presents the temporal profile of the set of samples. As it can be seen in these figures, the sample containing an intermediate Au loading was the one exhibiting the highest photocatalytic efficiency for overall water splitting, decreasing in activity for lower or higher Au contents. The existence of an optimal Au loading can be understood by the contribution of two opposite effects. On one hand, higher Au loading would play a positive effect, increasing the catalytic activity and light-harvesting role of Au. On the other hand, high loadings are unfavourable by increasing Au particle size and G surface coverage.

To determine if the remarkable overall water splitting of 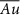/*ml*-G derives from the preparation procedure that is responsible for a strong Au-G interaction and the presence of facet oriented 111 Au nanoplatelets, control experiments using as photocatalysts *Au*/*ml*-G and *ml*-G were also performed. The results are also presented in Fig. 6.

It is worth noting that even in the absence of Au, *ml*-G has a residual overall water-splitting activity under ultraviolet irradiation. Importantly, comparison of the photocatalytic activity of 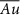/*ml*-G with that of an analogous sample containing unoriented Au NPs, *Au*/*ml*-G, shows that Au/*ml*-G exhibits even lower catalytic activity than *ml*-G. This indicates that in this sample prepared by adsorbing Au NPs on preformed G, unoriented Au NPs are disfavouring somewhat the overall water splitting and that the characteristic features of 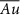/*ml*-G films what determines its remarkable photocatalytic activity in overall water splitting.

## Discussion

In view of prior characterization, we propose that the remarkable enhancement of photocatalytic activity for 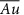/*ml*-G with respect to the other samples containing or not Au is the result of the one-step pyrolytic preparation procedure that produces a strong Au-G grafting and preferential (111) facet orientation of Au with nanoplatelet morphology. In the literature there is an ample number of precedents showing that graphenes in minor amounts can increase the photocatalytic activity of TiO_2_ and other metal oxide semiconductors, a fact that has been attributed to the increase of charge separation by electron migration from the semiconductor conduction band to the G (ref. 39). In recent precedents, multiple-step preparation of Au NPs without any preferential orientation deposited on G/TiO_2_ nanocomposites without strong grafting have reported H_2_ production rates around 1 mmol per g_composite_ per h under ultraviolet–visible irradiation in the presence of methanol as sacrificial electron donor^40^,^41^. In the present case, the strong interaction of oriented Au nanoplatelets and graphene can lead to an efficient charge separation disfavouring e^−^/h^+^ recombination by prompt migration of e^−^ to *ml*-G. The strong Au-G interaction is experimentally supported by the relatively small average particle size of Au nanoplatelets in spite of the prolonged treatment at elevated pyrolysis temperature (900 °C for 2 h), the shift in XPS of 1.4 eV in the Au 4f binding energy, the complete removal of N from G and occurrence of preferential morphology maximizing the contact area between nanoplatelets and graphene.

**Figure 7:**
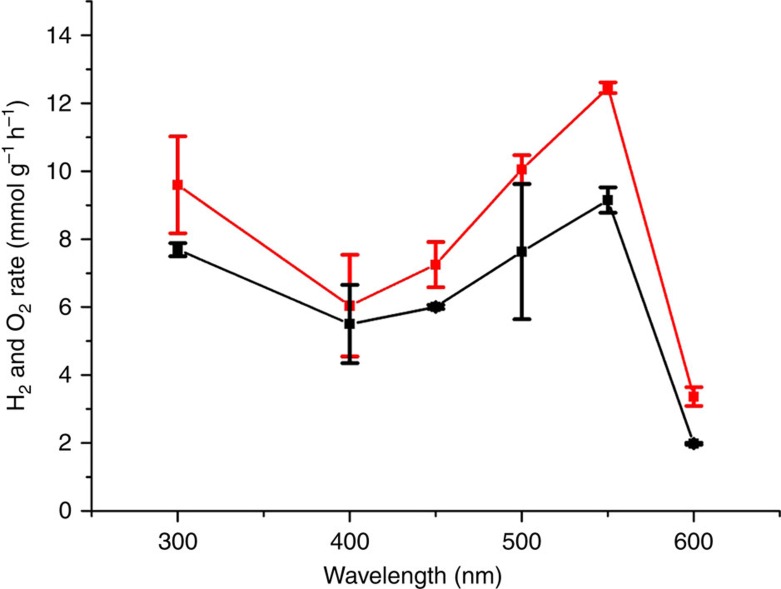
Photoresponse spectrum referred to the total photocatalyst amount. H_2_ (red) and O_2_ (black) production rates for 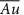
*/ml*-G (Au content 1 μg cm^−2^, total photocatalyst content 4.25 μg cm^−2^) with estimated errors (calculated as the square root of the sum of (a-ā)^2^, being a the value of the data set and ā the mean of the data set, divide by the number of data points) using monochromatic light (150-W Xe lamp). Irradiation time: 6 h. Note that the production rate is presented in mmol per g_composite_ per h due to the much lower power when irradiating with monochromatic light.

To gain understanding on the operation of the photocatalytic mechanism, and particularly the role of Au nanoplatelets, the photoaction spectrum for 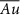/*ml*-G was determined by studying the overall water splitting activity of 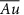/*ml*-G in the absence of any sacrificial donor using monochromatic light in the range from 300 to 600 nm. The results are presented in Fig. 7.

As it can be seen there, the photocatalytic activity has a clear relative minimum in the 350–450 nm exhibiting the maximum H_2_ generation activity at about 550 nm in the visible region. This photoaction spectrum of 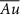/*ml*-G agrees very well with the plasmon band of Au nanoplatelets (Supplementary Fig. 7). Thus, the photoaction spectrum not only proves the photocatalytic activity of 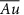/*ml*-G under visible light irradiation but also provides a strong support that Au nanoplatelets can act as light-harvesting units leading to charge separation. Moreover, a control using a 400-nm cutoff filter shows that *ml*-G in the absence of Au nanoplatelets does not exhibit photocatalytic activity in the visible region. This lack of visible light photocatalytic activity contrasts with the photocatalytic activity data shown in Fig. 6 for ultraviolet–visible light irradiation of *ml*-G that should be attributed to the ultraviolet zone of the irradiation light.

The use of monochromatic light allows determining the apparent quantum yield (Φ) for the overall water splitting that was 0.08% at 300 nm. Although this apparent Φ_300_ value is still low, it is similar to the one that reported for H_2_ evolution using Pt-containing g-C_3_N_4_ using triethanolamine as sacrificial electron donor^20^ and about two orders of magnitude higher than that for O_2_ evolution with the RuO_2_-modified g-C_3_N_4_ photocatalyst in the presence of Ag^+^ as sacrificial electron acceptor^20^. Note that in the present case, Φ_300_ 0.08% corresponds to the overall water splitting in the absence of any additive.

**Figure 8:**
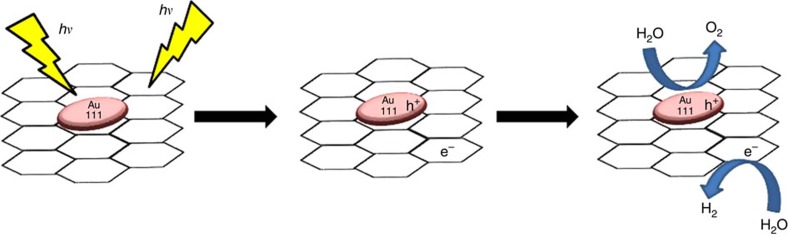
Proposed mechanism. Proposed mechanism for the photocatalytic reaction of water splitting.

Considering that *ml*-G exhibits also some residual for overall water splitting, a possible photocatalytic mechanism for ultraviolet–visible light irradiation of 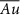/*ml*-G films is proposed in Fig. 8. In this Fig. 8, G would show two different roles. On one hand, G could present photocatalytic activity on absorption of ultraviolet photons, with fast e^−^/h^+^ recombination. On the other hand, visible light can only excite Au nanoplatelets, promoting charge separation with electron migration from Au nanoplatelets to G that in this case would act as enhancer of the charge separation.

To provide some evidence to this proposal, the energy of electrons in the valence band was measured by ultraviolet photoelectron spectroscopy (UPS) spectroscopy for 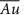/*ml*-G films. The valence band energy of 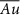/*ml*-G is presented in Supplementary Fig. 8 and it agrees with some minor changes with that previously reported for graphenes^42^. These minor changes could correspond to the levels modified or introduced by Au nanoplatelets. Extrapolation of the onset of valence band electrons gives an estimation of 1.5 V as the potential of holes in 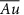/*ml*-G films. This value would make the process of H_2_O oxidation to O_2_ thermodynamically feasible.

In a different study trying to determine the location of h^+^, oxidation of Pb^2+^ to PbO_2_ was carried out in aqueous solution on 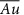/*ml*-G films using either Ce^IV^ and O_2_ as sacrificial electron acceptors. Subsequent determination of the presence of PbO_2_ by electron microscopy EDX analysis combined with EBSD and imaging by FESEM showed that the element Pb (EDX) in the crystal form corresponding to PbO_2_ (EBSD) was present on present on top of Au nanoplatelets, but Pb was below the detection limit on G. It should be, however, noted the different resolution of the microscopy techniques. Thus, while EDX has higher resolution and locates Pb on Au, electron diffraction has lower resolution than Au nanoplatelets. Methods section provides a detailed description of the experimental conditions, and Supplementary Figs 9 and 10 shows images indicating that oxidation occurs exclusively on the Au nanoplatelets, but not on the G surface.

In conclusion, herein it has been shown that films of nanometric thickness consisting in 111 facet-oriented Au nanoplatelets strongly grafted on *ml*-G are extremely efficient for the photocatalytic overall water splitting in to H_2_ and O_2_ in the absence of sacrificial electron donor with maximal production rate values of 1.2 mol_H2_ per g_composite_ per h. 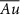/*ml*-G films exhibit even visible light photoresponse arising from the plasmon band of Au NPs. The estimated oxidation potential of valence band h^+^ is 1.5 V and electron microscopy shows the photodeposition of PbO_2_ exclusively on Au nanoplatelets. Further work should be aimed to gain deeper understanding on the interaction of Au nanoplatelets and G and to the optimization of the photocatalytic activity of these materials by alloying and doping.

## Methods

### Materials

All reagents used in this work were purchased from Sigma Aldrich and were used as received without further purification.

### Preparation of 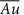 /*ml*-G films

The (111) oriented Au nanoparticles supported on *ml*-G film were prepared as reported before^29^. Briefly, an aqueous solution of chitosan (500 mg of chitosan in 25 ml of water) was spin coated onto 2 × 2 cm^2^ quartz substrates and dried. Quartz substrates were immersed into 1, 0.1 and 0.01 mM HAuCl_4_ aqueous solutions for 1 min to favour metal absorption on the film surface. After drying at 100 °C, the thin films were pyrolysed under Ar atmosphere at 900 °C.

### Estimation of the graphene mass in 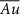 /*ml*-G films

The mass of graphene in nanometric 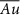/*ml*-G films was calculated following two alternative procedures:

On the basis of the weight of the chitosan precursor: the mass of an aqueous solution of chitosan (2 wt%) deposited onto a quartz substrate by spin coating was determined with a balance with 0.1 mg nominal precision by difference between the quartz substrate with and without the solution film. Thermogravimetric analysis shows that during the pyrolysis process at 900 °C a mass reduction of 60% takes place in the transformation of chitosan into graphene. Knowing the mass of solution deposited onto a 2 × 2-cm^2^ quartz substrate, the chitosan content (2 wt%) and the mass reduction in the pyrolysis, the weight of the residual graphene can be estimated by applying the equation 0.02 × 0.6 × mass of the solution. In this way, most of the experiments were performed with films containing an estimated mass of 13 μg of graphene in 2 × 2-cm^2^ films.

On the basis of density value and volume: estimated values of graphene density range between 1.5 and 2 g cm^−3^. Since our films are typically 4 cm^2^ and the film thickness determined by AFM was 20 nm, a volume of 8 × 10^−6^ cm^−3^ is calculated. By multiplying density per volume an estimation of the graphene mass can be obtained. Thus, these calculations estimate that the graphene mass in 2 × 2-cm^2^ films should be between 12 and 16 μg.

### HR-TEM images and EBSD

TEM images of an oriented 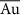
*/ml*-G sample were recorded at the Electron Microscopy Center of the Universitat de Valencia after abrasion of the quartz support by consecutive treatments consisting in mechanical polishing from the back side of the substrate until ∼100-μm thickness, followed by backside dimpling with a dimple grinder GATAN Model 656 and final low-angle, ion milling using an argon gun and plishing system Fishione Model 1,010. The fundamentals and detailed description of the methodology is described elsewhere^43^.

### UPS valence band measurement of 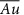 /*ml*-G

Ultraviolet photoemission measurements (UPS) were carried out in an ultra-high-vacuum ESCALAB 210 multianalysis system (base pressure 1.0 × 10^−10^ mbar) from Thermo VG Scientific. Photoelectrons were excited by means of a helium lamp by using the He II (40.8 eV) excitation lines. UPS spectra have been referred to the Fermi level (E_F). Previously to these measurements, samples were introduced in the analysis chamber and sputtered by using an Ar^+^ gun for 2 min, to clean the surface, removing adsorbates. Assignment of the energy peaks has been made based on the values previously reported in the literature^42^.

### Photodepostion of PbO_2_ by oxidation of Pb(OAc)_2_

A 1 × 1-cm^2^ 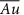/*ml*-G film was placed into a quartz cuvette. Then, 1.5 ml of a 1 mM aqueous solution Pb(OAc)_2_ and another 1.5 ml of a 1 mM aqueous solution of Ce(NH_4_)_2_(NO_3_)_6_ were introduced inside the cuvette. The cuvette was capped with a rubber septum and the aqueous phase purged with argon for 15 min before irradiation. The cuvette was irradiated with the Xe lamp for 1 h. After this time, the 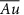/*ml*-G film was recovered, exhaustively washed with MilliQ water and studied by HRTEM and EDX to determine the location of Pb on the film.

In an additional experiment 1 × 1-cm^2^ 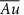/*ml*-G film was placed into a quartz cuvette adding 1.5 ml of a 1-mM aqueous solution Pb(OAc)_2_. The cuvette was open to the ambient and submitted to 3-h Xe lamp irradiation. After this time, the 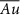/*ml*-G film was recovered, exhaustively washed with MilliQ water and studied by scanning electron microscopy, EDX and EBSD looking for the most intense peaks corresponding to PbO_2_, PbO and Pb. The images are presented in Supplementary Fig. 10.

### Photocatalytic hydrogen production tests

The 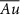/*ml*-G films were introduced in a close reactor and Ar-purged water was spread on top of the film until complete film coverage. Experiments with TEOA as sacrificial agent were carried out with Ar-purged water with a 15% w/v of TEOA. The H_2_ evolution tests were carried out in a 300-ml aluminium reactor with a quartz window connected to an Agilent 490 Micro GC (Molsieve 5A column with Ar as carrier gas) and irradiated with a 300-W Xe lamp. The experiments performed under monochromatic irradiation were carried out with a 150-W Xenon lamp through a Czerny Turner monochromator. The temperature and pressure inside the reactor was controlled through a thermocouple and a manometer, respectively.

### Data availability

All relevant data are available from the authors.

## Additional information

**How to cite this article:** Mateo, D. *et al*. 111 oriented gold nanoplatelets on multilayer graphene as visible light photocatalyst for overall water splitting. *Nat. Commun.* 7:11819 doi: 10.1038/ncomms11819 (2016).

## Supplementary Material

Supplementary Information Supplementary Figures 1-10
