# Supplementary material for: 111 oriented gold nanoplatelets on multilayer graphene as visible light photocatalyst for overall water splitting
Source: Nat Commun. 2016 Jun 6;7:11819. doi: 10.1038/ncomms11819 (PMC4897748; doi:10.1038/ncomms11819)
Supplement: Supplementary Information — Supplementary Figures 1-10 [file ncomms11819-s1.pdf]

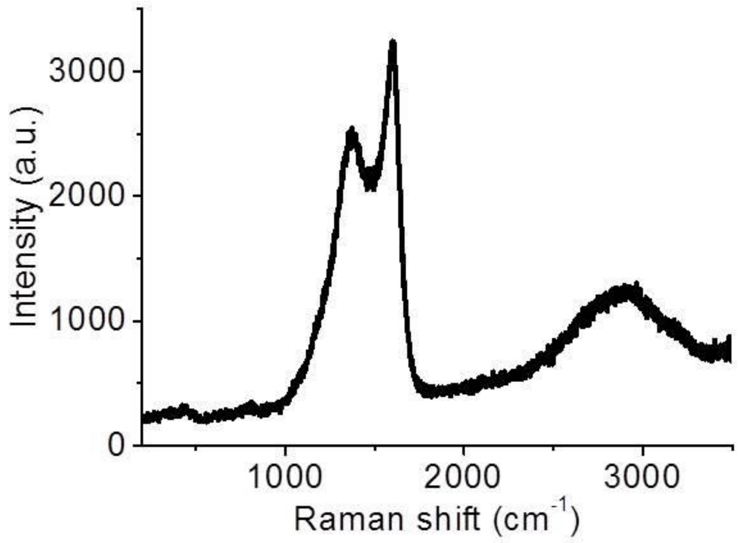

Supplementary Figure 1. Raman spectra of $\overline{Au}/ml$-G films. Raman excitation wavelength 512 nm.

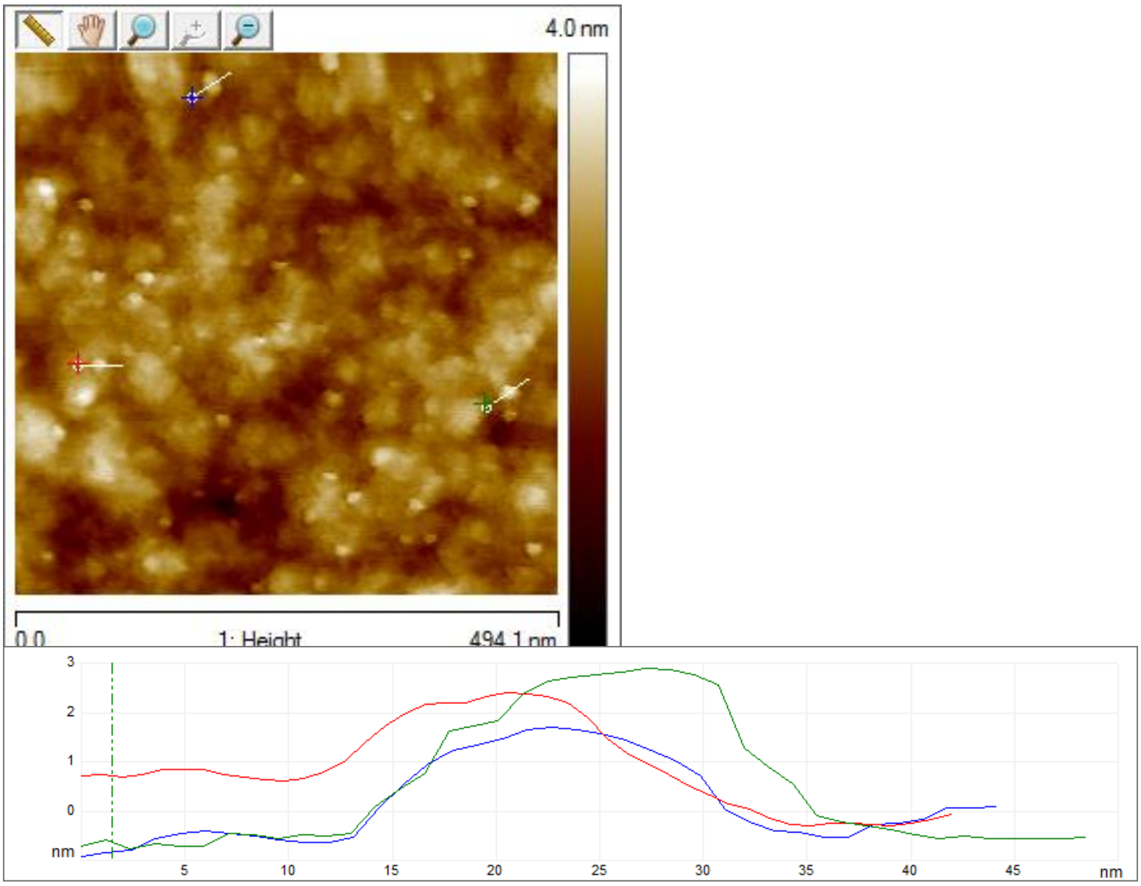

Supplementary Figure 2. AFM image of of $\overline{Au}/ml$-G film and measurement of some nanoplatelets.

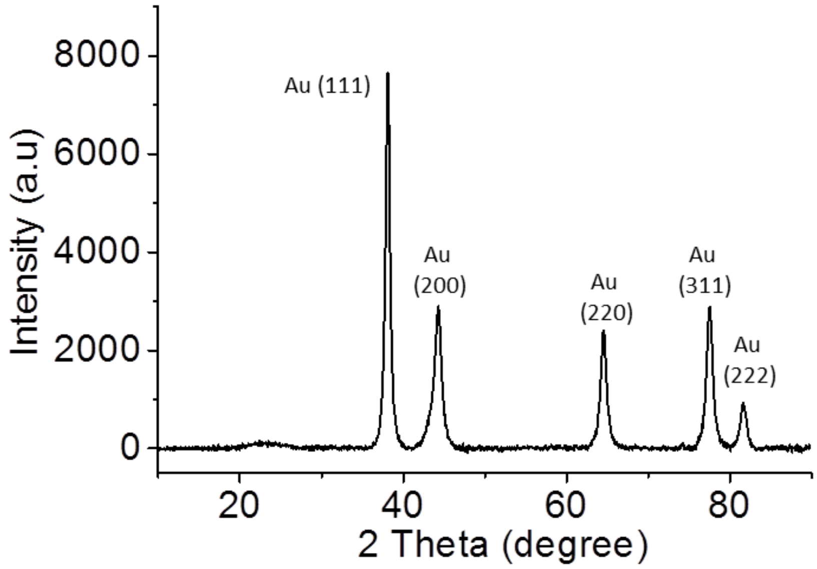

Supplementary Figure 3. XRD diffractogram of non-oriented Au NPs.

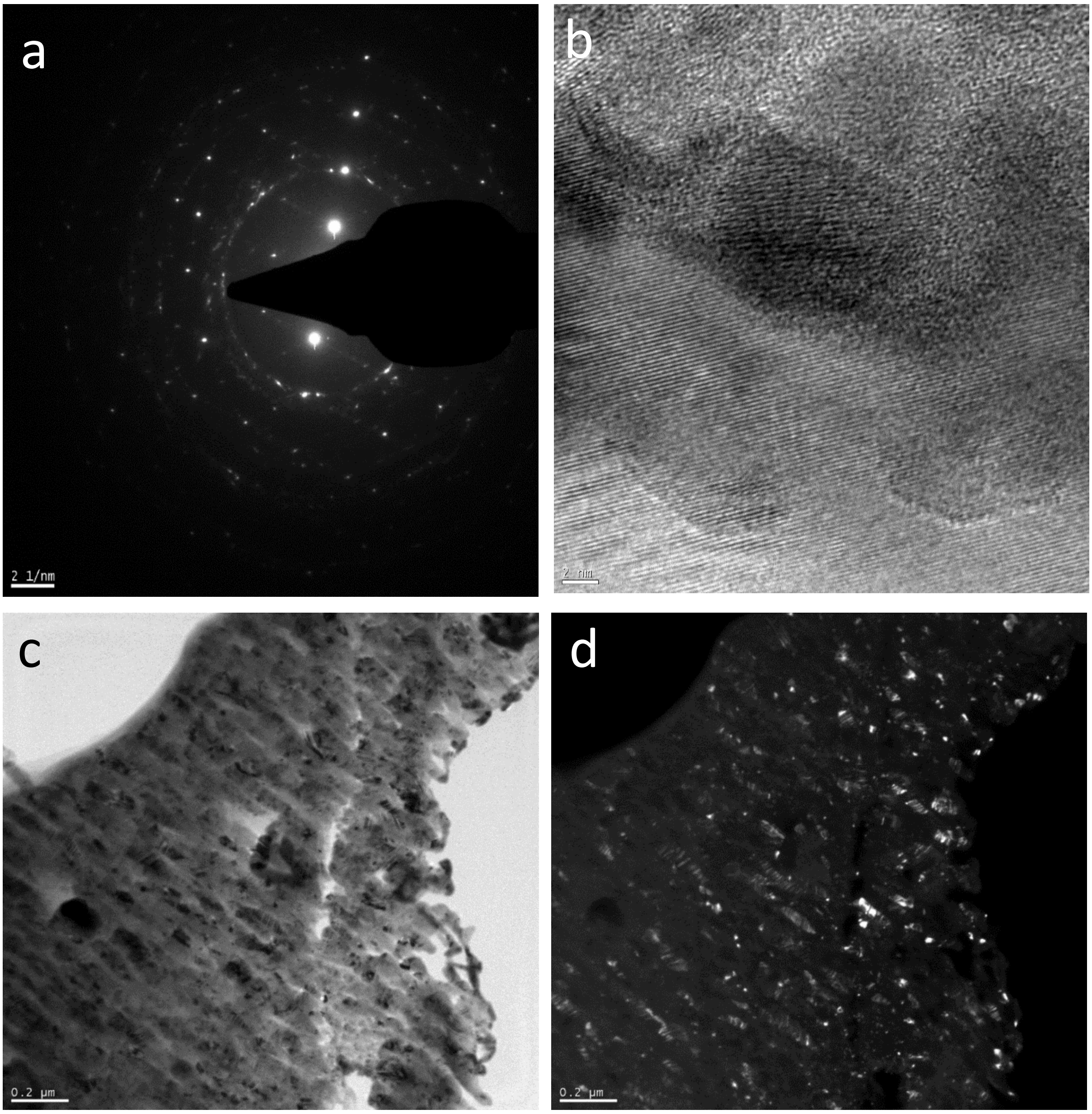

Supplementary Figure 4. Selected area electron diffraction (a) and TEM images (b, c and d) at two different magnifications of $\overline{Au}$/*ml*-G films. Images b shows the crystallinity of graphene.

Overall (c) and filtered 1. 1. 1 (d) facet orientation TEM images taken for strips of $\overline{Au}$/*ml*-G sample after removal of quartz substrate. Scale bar of 0.2 µm in all cases

a) b) c)

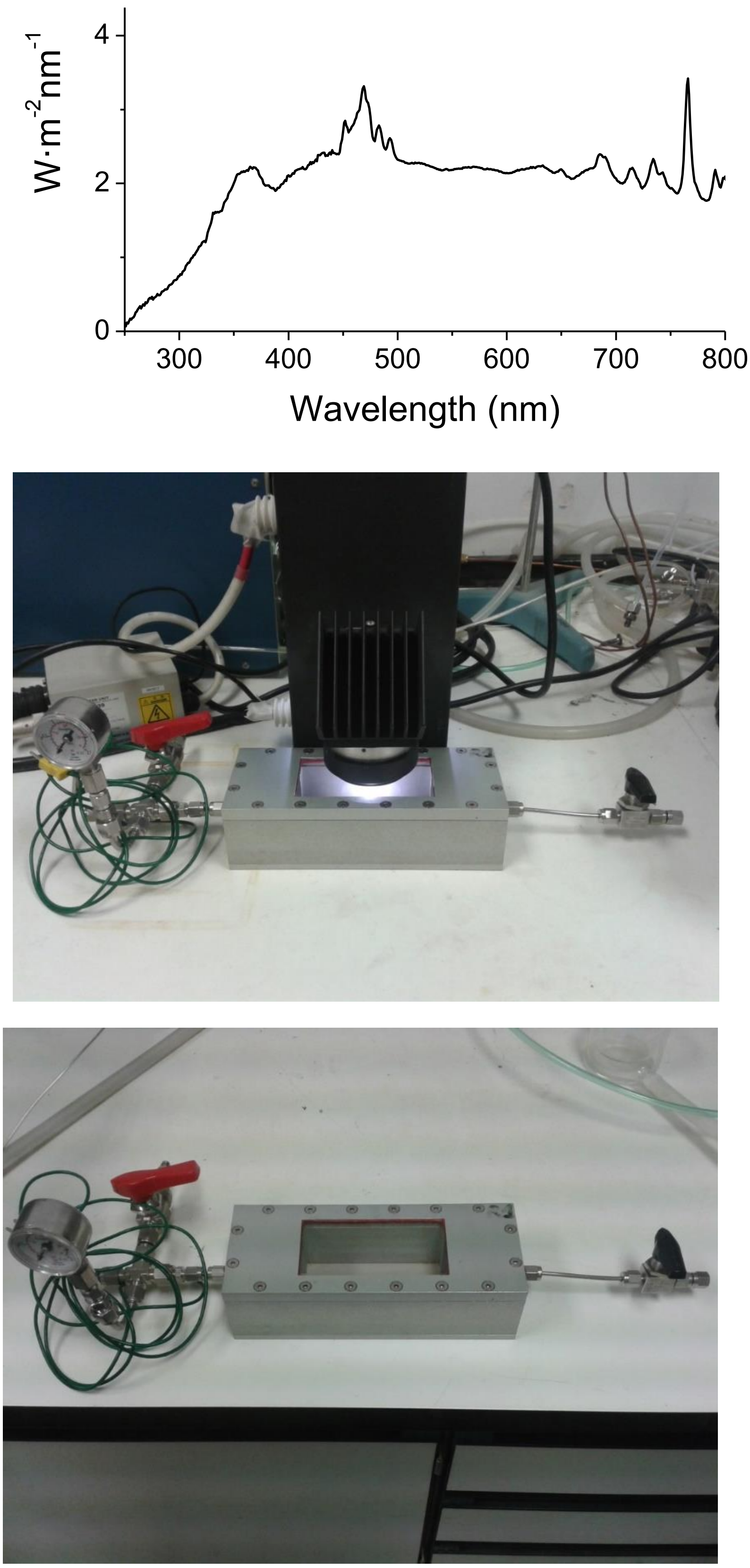

Supplementary Figure 5. Xenon lamp irradiation spectra (a) and photographs of the irradiation system (b) and the photo reactor (c).

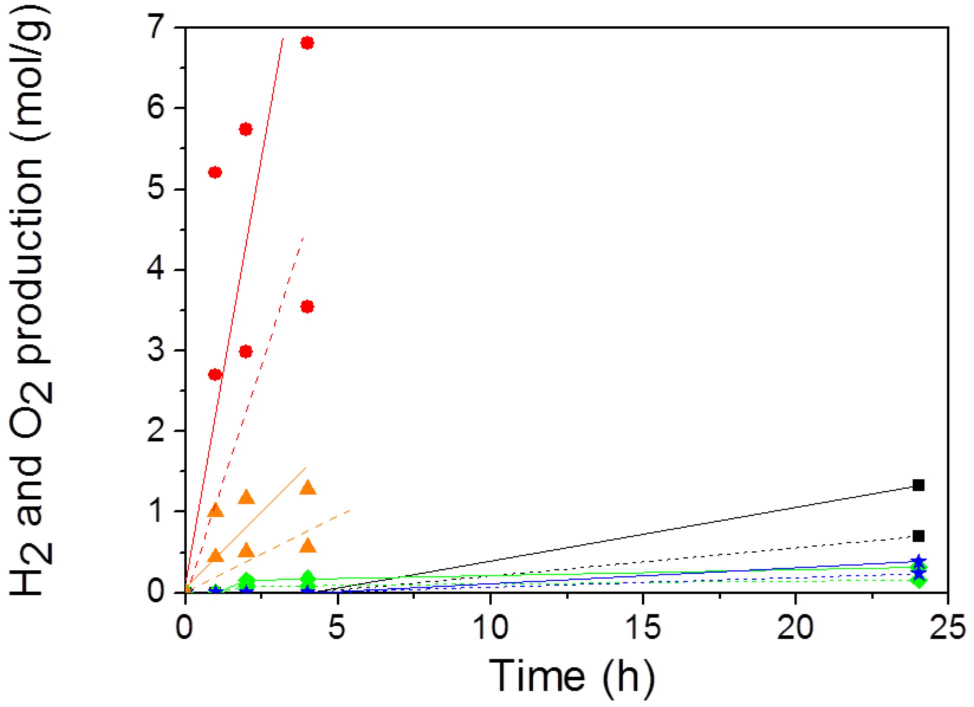

Supplementary Fig. 6. Photocatalytic H_2 (solid lines) and O_2 (dash lines) production in mols divided per total photocatalyst mass (Au plus G) for the studied samples: *ml*-G (orange) (G content 3.25 µg cm^{-2}), *Au/ml*-G (blue) (unoriented, Au content 30.6 µg, total photocatalyst content 30.6 mg), $\overline{Au}$/*ml*-G (black) (Au content 0.2 µg×cm^{-2}, total photocatalyst content 3.45 µg cm^{-2}), $\overline{Au}$/*ml*-G (red) (Au content 1 µg×cm^{-2}, total photocatalyst content 4.25 µg cm^{-2}) and $\overline{Au}$/*ml*-G (green) (Au content 13.5 µg×cm^{-2}, total photocatalyst content 16.75 µg cm^{-2}).

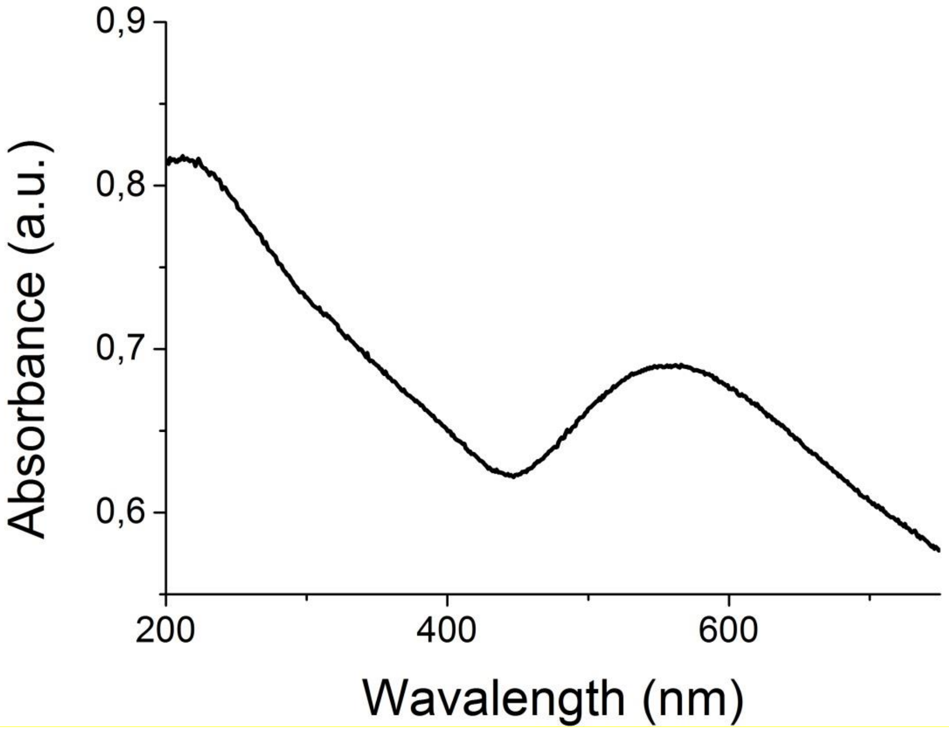

Supplementary Figure 7. UV-Vis spectra of a $\overline{Au}/ml$-G film.

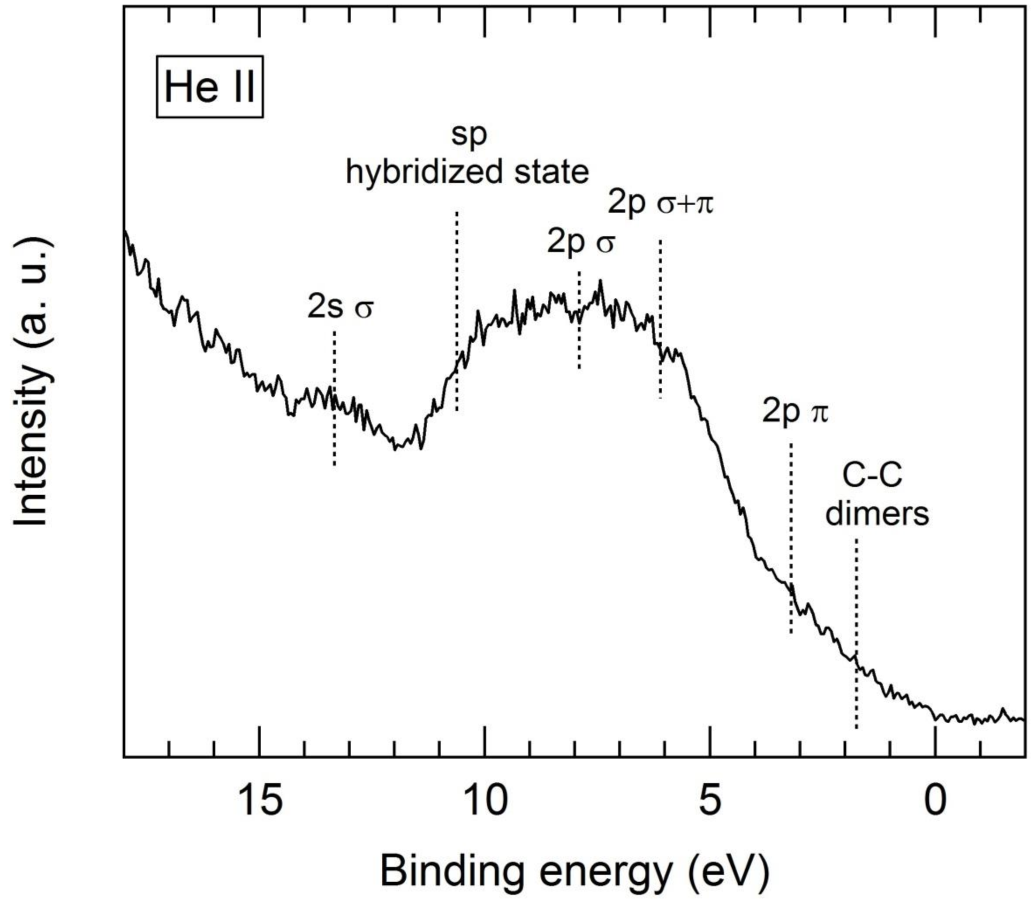

Supplementary Figure 8. Valence band structure of $\overline{Au}/ml$-G film measured with a He lamp (40 eV).

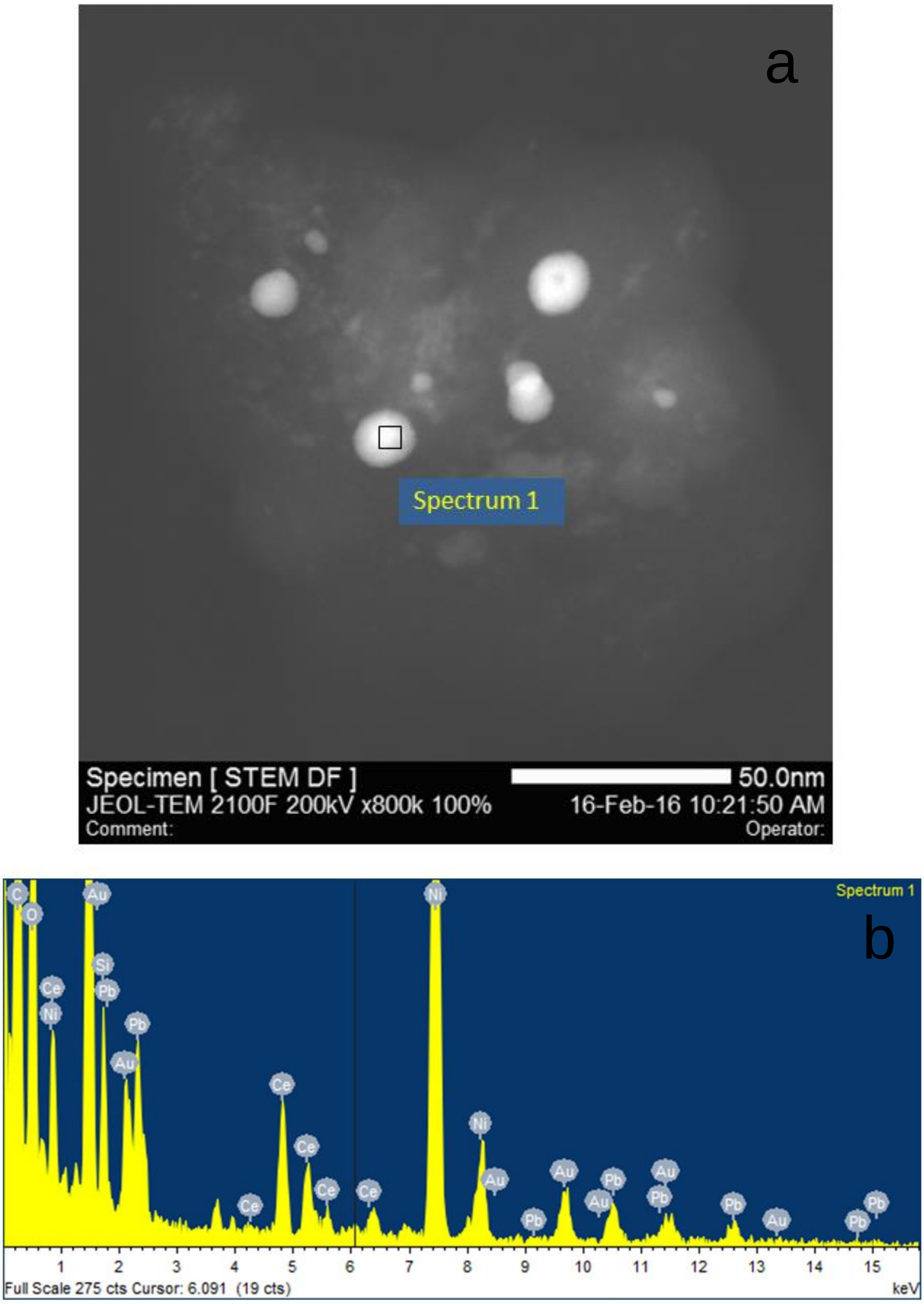

Supplementary Figure 9. Location of holes by mapping photodeposited Pb. STEM image of the $\overline{Au}$/*ml*-G film (a) and EDX spectrum of the locations marked in panel (b) after submitting the $\overline{Au}$/*ml*-G film to irradiation for 1 h in an aqueous solution containing $Ce(NH_4)(NO_3)_6$ as sacrificial electron acceptor and $Pb(OAc)_2$ as electron donor. Scale bar 50 nm. The images prove that Pb has been deposited on Au nanoplatelets. Analogous images show that Pb is not deposited on G.

A)

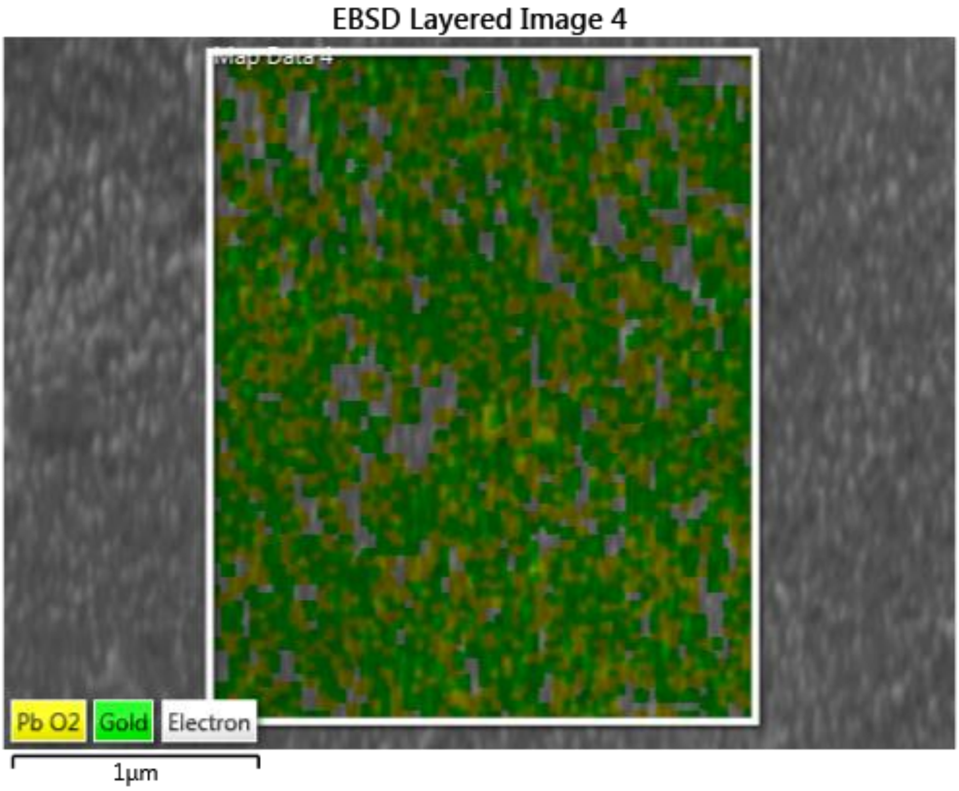
EBSD Layered Image 4
Map Data 4
Pb O2
Gold
Electron
1µm

B)

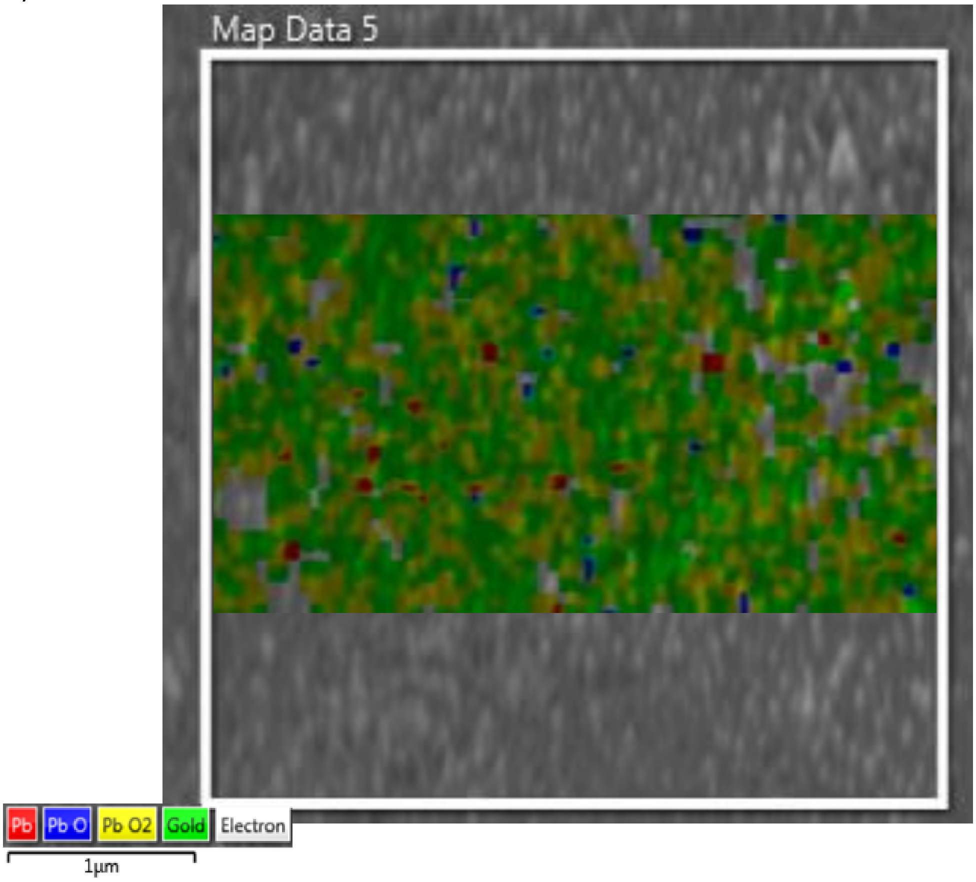
Map Data 5
Pb
Pb O
Pb O2
Gold
Electron
1µm

c)

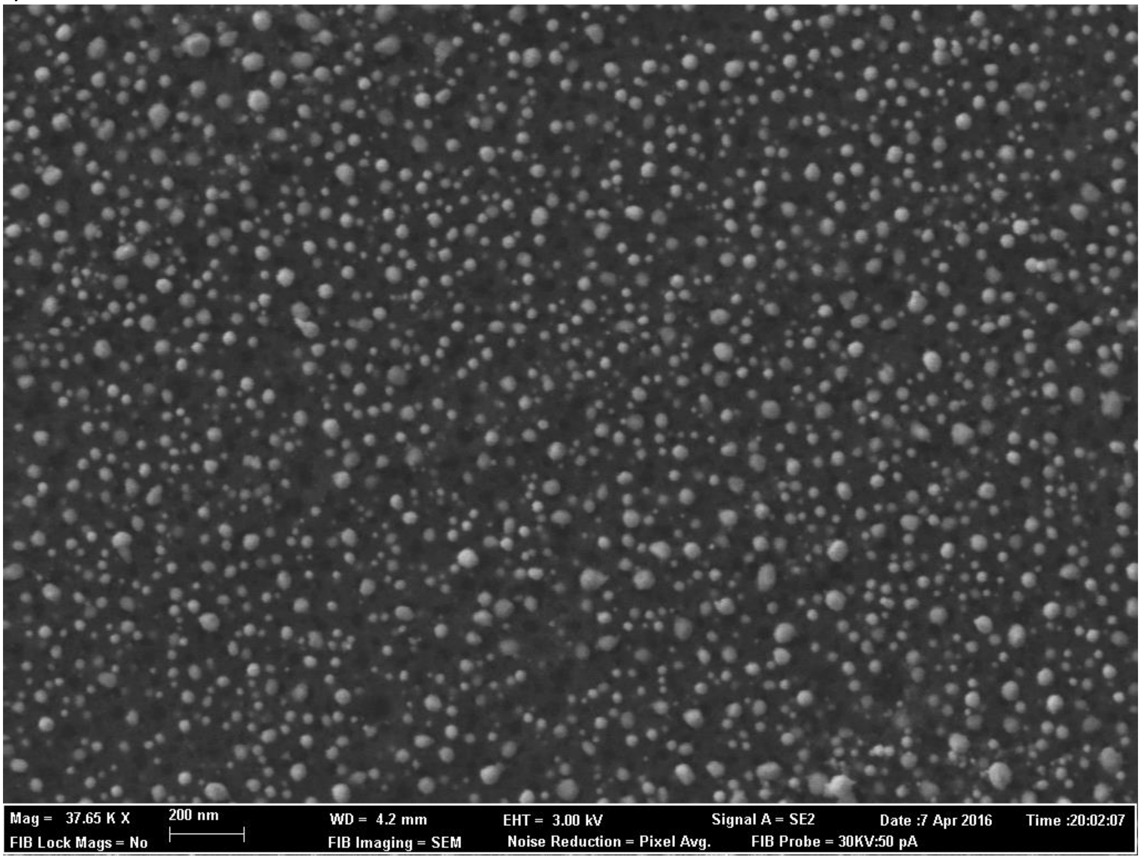

Supplementary Fig. 10. Oxidation state of photodeposited Pb. A) Superposition of SEM, elemental Au mapping and PbO2 diffraction. B) Another superposition of images corresponding to SEM, Au elemental mapping and diffraction peaks corresponding to PbO_2, PbO and Pb. For color codes see the Figures. C) FESEM image of the $\overline{Au}/ml$-G sample after irradiation in an aerated solution of $Pb(OAc)_2$ with Xe lamp for 3 h.
